# Help Wanted

**Published:** 1890-06

**Authors:** Edward T. Balch

**Affiliations:** South Bend, Pacific County, State of Washington


### HELP WANTED.

Maud R., æt. ten, demi-brunette, of healthy, vigorous parentage, about one and a half years ago, whilst playing with other children, fell forward and hurt herself in the chest. Parents thought it a trifling matter, thought it would get better anyway, consequently the case has not received any treatment whatever.

She is now brought to me, pale, emaciated, nervous, appetite capricious, does not care to play. Sleep disturbed by frightful dreams, during the day piteously begs her mother to take her, she is so afraid. She feels as though snakes were on her back. What is the remedy for the symptoms, sensation as though snakes were on back? She is now under Arnica, high. Suggestions thankfully received, and results acknowledged.

EDWARD T. BALCH, M. D.

SOUTH BEND, PACIFIC COUNTY, STATE OF WASHINGTON.

